# Space Detumbling Robot Arm Deployment Path Planning Based on Bi-FMT* Algorithm

**DOI:** 10.3390/mi12101231

**Published:** 2021-10-10

**Authors:** Ning Chen, Yasheng Zhang, Wenhua Cheng

**Affiliations:** 1 Department of Graduate Management, Space Engineering University, Beijing 101416, China; 18811021510@163.com; 2 Department of Space Command, Space Engineering University, Beijing 101416, China; zyspublic@163.com

**Keywords:** space detumbling robot, path planning, ADAMS, Bi-FMT*

## Abstract

In order to avoid damage to service satellites and targets during space missions and improve safety and reliability, it is necessary to study how to eliminate or reduce the rotation of targets. This paper focused on a space detumbling robot and studied the space detumbling robot dynamics and robot arm deployment path planning. Firstly, a certain space detumbling robot with a ‘platform + manipulator + end effector’ configuration is proposed. By considering the end effector as a translational joint, the entire space detumbling robot is equivalent to a link system containing six rotating joints and three translational joints, and the detailed derivation process of the kinematic and dynamic model is presented. Then, ADAMS and MATLAB were used to simulate the model, and the MATLAB results were compared with the ADAMS results to verify the correctness of the model. After that, the robot arm deployment problem was analyzed in detail from the aspects of problem description, constraint analysis and algorithm implementation. An algorithm of robot arm deployment path planning based on the Bi-FMT* algorithm is proposed, and the effectiveness of the algorithm is verified by simulation.

## 1. Introduction

Space operation and control refer to the on-orbit activity for specific targets with or without people’s participation to achieve proximity detection, auxiliary orbit maneuvers, fault maintenance, fuel filling, system upgrades, assembly, construction, rescue and space debris removal [1]. From the Lunakod/Luna project of the Soviet Union and the SAINT (Satellite Inspector) project of the US to Phoenix and SMART-OLEV, the development of space operation and control has always been promoted by the Space Age. A series of experiments have been carried out to develop and verify relevant technologies by the space powers of the world. Space operation and control have become an important indicator of a country’s space force. A review of the development of space operation and control projects around the world is summarized in [2]. Space robot motion and control are the core of space operation and control.

Whether it is on-orbit service (OOS), on-orbit assembly or space debris removal, the approach to non-cooperative targets is important. These non-cooperative targets usually have complex attitude movements, including spins, precession and tumbling, which greatly affect the approach process. In order to avoid adverse damage to service satellites and targets during operation and improve safety and reliability, it is necessary to study how to eliminate or reduce the rotation of the target. Generally speaking, as long as the relative state of the service satellite and target meets certain requirements, effective acquisition can be achieved. From the perspective of detumbling, detumbling can be divided into relative detumbling and absolute detumbling. Relative detumbling means that it does not change the target’s motion state but uses the service satellite’s own adjustment capabilities to change its own motion state to meet relative state constraints. For example, approaching from the target spin axis direction [3] is a typical relative detumbling strategy.

This article focused on absolute detumbling, that is, through the direct or indirect interaction between the service satellite and target, the target state is changed to satisfy the capture condition. In principle, the main operation to make the target detumble is to apply additional torque to the target. According to the different torque sources, absolute detumbling can be divided into contact detumbling and non-contact detumbling. A series of technical verification tests was conducted by the space powers, proposing numerous detumbling methods. These methods are shown in Table 1.

Considering technical maturity and energy consumption, among these methods, the robotic contact method is the most feasible to implement and verify. Additionally, this method combines capture and detumbling together which is very suitable for OOS. As one of the key technologies in space robot control, path planning generates a motion sequence to guide the robot from the initial state to the goal state safely. Path planning is widely used in the field of robotics and has accumulated a wide range of research results [29,30]. Roughly, path planning can be divided into two categories: complete planning and sampling-based planning. 

Complete path planning is usually planned directly in the state space, with the Depth First Search (DFS), Breadth First Search (BFS) and Dijstra algorithms representing the original algorithms, and the Astar algorithm representing the most commonly used algorithm. The advantage of this method is that it can completely obtain the solution, but the cost is that the algorithm will become very complicated. This cost is not obvious in path planning in low-dimensional spaces but becomes very prominent in high-dimensional spaces. Since the actual work space-to-state space mapping is non-linear, it is very troublesome to represent obstacles and constraints in the state space. The usual approach is to discretize the space and detect the discrete parts. However, as mentioned earlier, this type of discretization is fine in low-dimensional spaces, but it will bring unimaginable complex calculations in high-dimensional spaces, which directly promotes the generation of sampling-based path planning algorithms.

Sampling-based path planning generally does not plan directly in the state space but randomly arranges a certain density of the sample space to approximate the state space. Sampling-based path planning is also divided into two types: One is graph-based, which scatters sampling points in the original state space and extracts the path by connecting those points with consideration of constraints, such as the probabilistic road map (PRM) algorithm and its improvement. The other is tree-based, which randomly arranges a point in the state space and iteratively grows the tree with the purpose of connecting the starting and ending points, such as the rapid exploration random tree (RRT) algorithm and its improvement. Whether graph-based or tree-based, these algorithms do not need to consider the distribution of obstacles in space but only need to perform collision detection on random sampling points. The planning speed is quite fast and can be used in any dimensional space, and, in particular, path planning has been widely used in high-dimensional spaces.

Due to the complexity of characterizing obstacles and constraints in the state space, complete planning is usually limited to handling low-dimensional problems with simple-shaped obstacles. Sampling-based planning does not need to express obstacles and constraints explicitly but instead combines search-based sampling and performs safety verification through a collision detection algorithm. By separating the planning problem from the actual physical and geometric problems, sampling-based planning greatly accelerates the speed of planning, especially in high-dimensional problems with complex-shaped obstacles. 

Space detumbling is a multi-disciplinary complex system engineering problem involving basic disciplines such as mathematics, physics and materials and combining technical disciplines such as control, computer and simulation. Measurement noise, actuator noise, high-order dynamics and orbital perturbations all contribute to the complexity and uncertainty of space detumbling. Considering the uncertainty in space robot motion and control, robot platforms need to have near real-time planning ability in order to handle various uncertainties quickly and safely. Now, the solution for handling uncertainty is mainly divided into three categories. One is to optimize the design of a new spatial structure, as in [31,32]; another is to change the way of thinking and decompose the problem reasonably and abstractly, as explored by Kumar et al., where they decomposed any 3D motion into a 3D translation and three rotations about specific axes related to the object, which allows planning for 3D dexterous in-hand manipulation with a moderate increase in complexity in just a few seconds [33]; the third is to use probabilistic analysis methods. Sampling-based path planning achieves an optimal solution under probability analysis through a reduction in constraints and backward detection and evaluation, which can not only ensure the calculation efficiency but also deal with various constraints well. 

Commonly used sampling-based path planning algorithms include PRM [34], RRT [35] and EST [36]. These algorithms can quickly find a feasible path, especially in high-dimensional spaces. However, when the sampling points are too few or the distribution is unreasonable, sampling-based path planning only obtains a feasible path, not the optimal path. In order to solve this problem, scholars have proposed asymptotically optimal versions, PRM* [37] and RRT* [38], where, as the number of samples increases, the solution path obtained will inevitably converge to a global optimum, as with BIT* [39] and RRT# [40]. It is particularly worth noting that the fast marching tree (FMT*) algorithm proposed by Janson et al. [41] is a conceptually novel sampling-based path planning algorithm, and numerical simulation experiments have shown that the FMT* algorithm can converge to the optimal solution faster than PRM* and RRT* in the face of a high-dimensional state space and complex collision detection. 

Although sampling-based path planning has not been applied in space missions, its effects and advantages for solving problems with high dynamics and uncertain environments have been verified in ground practical systems. In the Urban Challenge held by the Defense Advanced Research Projects Agency (DARPA), almost all of the winners adopted sampling-based path planning [42,43,44,45]. Since the path planning framework is universal, it seems that those research results can be applied to space path planning in theory. However, spacecraft motion is very different from ground robots, especially in space mapping and the C-space [46,47,48,49], meaning these planners cannot be directly applied to space missions without modification. Some scholars have studied the feasibility of sampling-based path planning in space missions [50,51,52,53], especially the studies by Starek et al. [54,55,56,57], in which the real-time implementability, safety and propellant efficiency of path planning by using FMT* or Bi-FMT* were thoroughly discussed in detail.

In the early stage, an improved sampling-based approach for spacecraft proximity operation path planning under Clohessy–Wiltshire–Hill dynamics based on a modified version of the FMT* algorithm with a safety strategy was proposed and analyzed in [58]. In this work, the dynamics and robot arm deployment path planning problem of a certain space detumbling robot were analyzed. Section 2 introduces the design and structure of the space detumbling robot. The kinematics and dynamics of the robot are also analyzed in this section. Then, the detumbling robot arm deployment path planning by using the Bi-FMT* algorithm is described based on the prevention model in detail from the aspects of problem description, constraint analysis and algorithm implementation in Section 3. Additionally, the proposed approach is illustrated by using two numerical experiments in Section 4. Finally, the conclusion and future work directions are provided in Section 5.

## 2. Space Detumbling Robot Modeling

**Figure 1:**
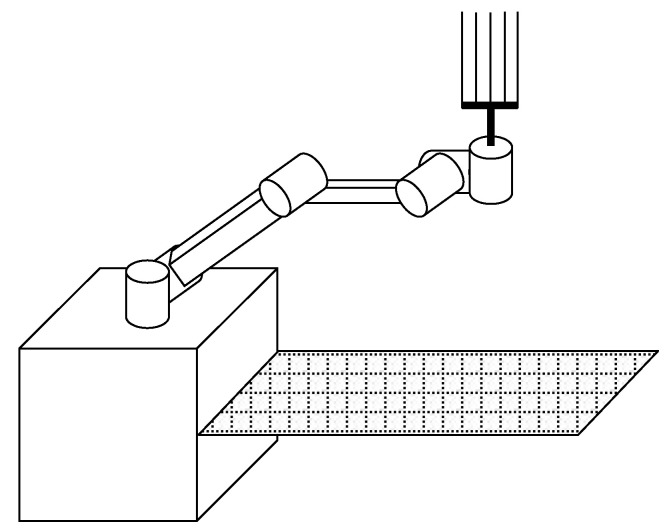
The space detumbling robot designed in this paper.

The space detumbling robot designed in this paper is shown in Figure 1. As shown in the figure, the robot is divided into three parts: satellite platform, manipulator and end effector. Among them, the satellite platform has a ‘central rigid body + solar panel’ configuration. The arm adopts the configuration of ‘elbow-shaped mechanical arm + spherical wrist’. Additionally, the end effector is designed as a flexible brush, which can be considered equivalent to a translational joint with a fixed length.

### 2.1. Kinematics

Generally, a robot arm system can be treated as a series of links connected by joints. These joints can be divided into single-degree-of-freedom joints and multi-degree-of-freedom joints. In fact, multi-degree-of-freedom joints can also be considered as continuous single-degree-of-freedom joints connected by a zero-length link. Therefore, in this study, it is assumed all joints are single-degree-of-freedom joints. Each coordinate system satisfies the Denavit–Hartenberg (DH) convention, that is,

(1) O_i+1_X_i+1_ is perpendicular to O_i_Z_i_;
(2) O_i+1_X_i+1_ intersects with O_i_Z_i_.

Under the condition of the DH convention, the transformation matrix can be expressed as the product of four basic matrices [59]:

(1) $$\begin{array}{c} A=R\left(z,\theta \right)T\left(z,d\right)T\left(x,a\right)R\left(x,\alpha \right)\\ \;\;=\left[\begin{array}{cccc} \cos\theta & \sin\theta & 0 & 0\\ -\sin\theta & \cos\theta & 0 & 0\\ 0 & 0 & 1 & 0\\ 0 & 0 & 0 & 1 \end{array}\right]\left[\begin{array}{cccc} 1 & 0 & 0 & 0\\ 0 & 1 & 0 & 0\\ 0 & 0 & 1 & d\\ 0 & 0 & 0 & 1 \end{array}\right]\left[\begin{array}{cccc} 1 & 0 & 0 & a\\ 0 & 1 & 0 & 0\\ 0 & 0 & 1 & 0\\ 0 & 0 & 0 & 1 \end{array}\right]\left[\begin{array}{cccc} 1 & 0 & 0 & 0\\ 0 & \cos\alpha & \sin\alpha & 0\\ 0 & -\sin\alpha & \cos\alpha & 0\\ 0 & 0 & 0 & 1 \end{array}\right]\\ \;\;=\left[\begin{array}{cccc} \cos\theta & \sin\theta \cos\alpha & \sin\theta \sin\alpha & a\cos\theta \\ -\sin\theta & \cos\theta \cos\alpha & \cos\theta \sin\alpha & -a\sin\theta \\ 0 & -\sin\alpha & \cos\alpha & d\\ 0 & 0 & 0 & 1 \end{array}\right] \end{array}$$

where *θ* represents the angle from O_i_X_i_ to O_i+1_X_i+1_ measured in a plane perpendicular to O_i_Z_i_; *d* represents the distance from the origin O_i_ to the intersection of O_i+1_X_i+1_ and O_i_Z_i_; *a* represents the distance between O_i_Z_i_ and O_i+1_Z_i+1_ measured along O_i+1_X_i+1_; *α* represents the angle measured from O_i_Z_i_ to O_i+1_Z_i+1_ in the plane perpendicular to O_i+1_X_i+1_.

Then, the transformation matrix of O_n+1_-X_n+1_Y_n+1_Z_n+1_ relative to O_1_-X_1_Y_1_Z_1_ is

(2) $$A_{n+1}^{1}=A_{1}A_{2}\cdots A_{n}=\prod_{i=1}^{n}\left[\begin{array}{cccc} \cos\theta_{i} & \sin\theta_{i}\cos\alpha_{i} & \sin\theta_{i}\sin\alpha_{i} & a_{i}\cos\theta_{i}\\ -\sin\theta_{i} & \cos\theta_{i}\cos\alpha_{i} & \cos\theta_{i}\sin\alpha_{i} & -a_{i}\sin\theta_{i}\\ 0 & -\sin\alpha_{i} & \cos\alpha_{i} & d_{i}\\ 0 & 0 & 0 & 1 \end{array}\right]=\left[\begin{array}{cc} R_{n+1}^{1} & T_{n+1}^{1}\\ 0 & 1 \end{array}\right]$$

Suppose the angular velocity of end effector is *ω* and the linear velocity is *v*, and let

(3) $$\begin{array}{l} \left[\omega_{n+1}^{1}\times \right]={\dot{R}}_{n+1}^{1}{\left(R_{n+1}^{1}\right)}^{T}\\ v_{n+1}^{1}={\dot{T}}_{n+1}^{1} \end{array}$$

From the transformation matrix, we can obtain

(4) $$\begin{array}{l} R_{i+1}^{1}=R_{i}^{1}R_{i+1}^{i}\\ T_{i+1}^{1}=R_{i}^{1}T_{i+1}^{i}+T_{i}^{1} \end{array}$$

Taking the joint variable *q_i_* as variable, by the chain rule, we can obtain

(5) $$\begin{array}{c} \left[\omega_{n+1}^{1}\times \right]={\dot{R}}_{n+1}^{1}{\left(R_{n+1}^{1}\right)}^{T}=\left(\sum_{i=1}^{n}\frac{\partial R_{n+1}^{1}}{\partial q_{i}}{\dot{q}}_{i}\right){\left(R_{n+1}^{1}\right)}^{T}=\left(\sum_{i=1}^{n}\frac{\partial \left(R_{n}^{1}R_{n+1}^{n}\right)}{\partial q_{i}}{\dot{q}}_{i}\right){\left(R_{n}^{1}R_{n+1}^{n}\right)}^{T}\\ \;\;\;\;=\left(\sum_{i=1}^{n}\left(\frac{\partial \left(R_{n}^{1}\right)}{\partial q_{i}}R_{n+1}^{n}{\dot{q}}_{i}+R_{n}^{1}\frac{\partial \left(R_{n+1}^{n}\right)}{\partial q_{i}}{\dot{q}}_{i}\right)\right){\left(R_{n+1}^{n}\right)}^{T}{\left(R_{n}^{1}\right)}^{T}\\ \;\;\;\;=\sum_{i=1}^{n}\left(\frac{\partial \left(R_{n}^{1}\right)}{\partial q_{i}}{\dot{q}}_{i}\right)R_{n+1}^{n}{\left(R_{n+1}^{n}\right)}^{T}{\left(R_{n}^{1}\right)}^{T}+R_{n}^{1}\sum_{i=1}^{n}\left(\frac{\partial \left(R_{n+1}^{n}\right)}{\partial q_{i}}{\dot{q}}_{i}\right){\left(R_{n+1}^{n}\right)}^{T}{\left(R_{n}^{1}\right)}^{T}\\ \;\;\;\;=\left[\omega_{n}^{1}\times \right]+R_{n}^{1}\left[\omega_{n+1}^{n}\times \right]{\left(R_{n}^{1}\right)}^{T}=\cdots \\ \;\;\;\;=\sum_{i=1}^{n}R_{i}^{1}\left[\omega_{i+1}^{i}\times \right]{\left(R_{i}^{1}\right)}^{T}\\ \;\;\;\;=\sum_{i=1}^{n}\left[\left(R_{i}^{1}\omega_{i+1}^{i}\right)\times \right]=\left[\left(\sum_{i=1}^{n}R_{i}^{1}\omega_{i+1}^{i}\right)\times \right]=\left[\left(\sum_{i=1}^{n}R_{i}^{1}\omega_{i}\right)\times \right] \end{array}$$

Therefore, the angular velocity of the end effector coordinate system relative to the fixed coordinate system is

(6) $$\omega_{n+1}^{1}=\sum_{i=1}^{n}R_{i}^{1}\omega_{i}$$

Similarly, the velocity of the end effector coordinate system relative to the fixed coordinate system is

(7) $$\begin{array}{c} v_{n+1}^{1}={\dot{T}}_{n+1}^{1}=\sum_{i=1}^{n}\frac{\partial T_{n+1}^{1}}{\partial q_{i}}{\dot{q}}_{i}=\sum_{i=1}^{n}\frac{\partial \left(R_{n}^{1}T_{n+1}^{n}+T_{n}^{1}\right)}{\partial q_{i}}{\dot{q}}_{i}=\sum_{i=1}^{n}\left(\frac{\partial \left(R_{n}^{1}T_{n+1}^{n}\right)}{\partial q_{i}}{\dot{q}}_{i}+\frac{\partial \left(T_{n}^{1}\right)}{\partial q_{i}}{\dot{q}}_{i}\right)\\ \;\;=\sum_{i=1}^{n}\left(\frac{\partial \left(R_{n}^{1}\right)}{\partial q_{i}}T_{n+1}^{n}{\dot{q}}_{i}+R_{n}^{1}\frac{\partial \left(T_{n+1}^{n}\right)}{\partial q_{i}}{\dot{q}}_{i}\right)+\sum_{i=1}^{n}\left(\frac{\partial \left(T_{n}^{1}\right)}{\partial q_{i}}{\dot{q}}_{i}\right)\\ \;\;=\sum_{i=1}^{n}\left(\frac{\partial \left(R_{n}^{1}\right)}{\partial q_{i}}{\dot{q}}_{i}\right)T_{n+1}^{n}+R_{n}^{1}\sum_{i=1}^{n}\left(\frac{\partial \left(T_{n+1}^{n}\right)}{\partial q_{i}}{\dot{q}}_{i}\right)+\sum_{i=1}^{n}\left(\frac{\partial \left(T_{n}^{1}\right)}{\partial q_{i}}{\dot{q}}_{i}\right)=\cdots \\ \;\;=\sum_{j=1}^{n}\left(\left[\omega_{j}^{1}\times \right]R_{j}^{1}T_{j+1}^{j}\right)+\sum_{j=1}^{n}\left(R_{j}^{1}\sum_{i=1}^{j}\left(\frac{\partial \left(T_{j+1}^{j}\right)}{\partial q_{i}}{\dot{q}}_{i}\right)\right)\\ \;\;=\sum_{i=1}^{n}\left(\left[\omega_{i}^{1}\times \right]R_{i}^{1}T_{i+1}^{i}\right)+\sum_{i=1}^{n}\left(R_{i}^{1}v_{i}\right)\\ \;\;=\sum_{i=2}^{n}\left(\left(\sum_{j=1}^{i-1}R_{j}^{1}\left[\omega_{j+1}^{j}\times \right]{\left(R_{j}^{1}\right)}^{T}\right)R_{i}^{1}T_{i+1}^{i}\right)+\sum_{i=1}^{n}\left(R_{i}^{1}v_{i}\right)\\ \;\;=\sum_{i=1}^{n}\left(\left(R_{i}^{1}\left[k\times \right]{\left(R_{i}^{1}\right)}^{T}\right)\left(R_{i+1}^{1}T_{i+1}^{i}+\cdots +R_{n}^{1}T_{n+1}^{n}\right)\omega_{i}\right)+\sum_{i=1}^{n}\left(R_{i}^{1}v_{i}\right)\\ \;\;=\sum_{i=1}^{n}\left(\left[\left(R_{i}^{1}k\right)\times \right]\left(T_{n+1}^{1}-T_{i}^{1}\right)\omega_{i}\right)+\sum_{i=1}^{n}\left(R_{i}^{1}v_{i}\right) \end{array}$$

which is

(8) $$\begin{array}{c} \left(\begin{array}{c} v_{n+1}^{1}\\ \omega_{n+1}^{1} \end{array}\right)=\left(\begin{array}{c} \sum_{i=1}^{n}\left(\left[\left(R_{i}^{1}k\right)\times \right]\left(T_{n+1}^{1}-T_{i}^{1}\right)\omega_{i}\right)+\sum_{i=1}^{n}\left(R_{i}^{1}v_{i}\right)\\ \sum_{i=1}^{n}R_{i}^{1}\omega_{i} \end{array}\right)\\ \;\;\;\;\;=\left(\begin{array}{c} J_{v}^{n+1}\\ J_{\omega }^{n+1} \end{array}\right)\dot{q}=J_{n+1}^{1}\dot{q} \end{array}$$

where *J* is the Jacobian matrix of the robot arm. When the joint is a revolute joint, *v_i_* is 0, and *ω_i_* is the angular velocity of the joint; when the joint is a translational joint, *ω_i_* is 0, and *v_i_* is the translational velocity of the joint.

**Figure 2:**
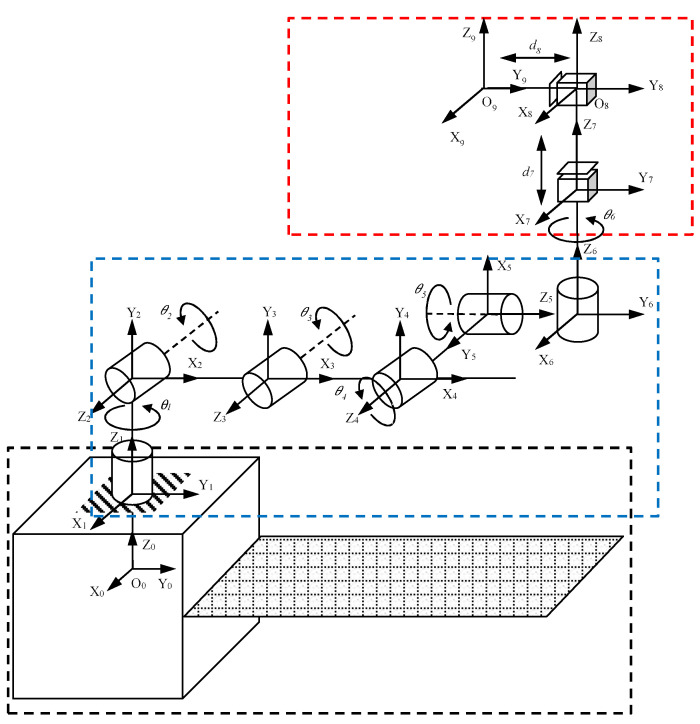
Space detumbling robot modeling.

By considering the end effector as a translational joint, the entire space detumbling robot is equivalent to a link system containing six rotating joints and three translational joints, as shown in Figure 2.

O_i_-X_i_Y_i_Z_i_ (i = 0, 1, …, 8) is the coordinate system of each link, which is fixed at the center of mass (CM) of the joint and meets the DH convention. Combined with the structure diagram presented in Figure 2, the DH parameters of the robot system can be obtained, as shown in Table 2. 

d_0_ is the distance from the platform CM to the center of Joint #1; L1 and L2 are the lengths of the two links of the elbow manipulator; d_6_ is the distance from the end effector to the center of the mounting flange of Joint #6; d_7_ and *a*_8_ are the vertical and horizontal displacements of the flexible brush.

As each joint of the robot arm is a rotating joint,

(9) $$\omega_{i}=\left(\begin{array}{c} 0\\ 0\\ 1 \end{array}\right)\omega_{i}$$

Therefore, the velocity and angular velocity of the flexible brush end coordinate system relative to the platform coordinate system are

(10) $$\begin{array}{c} \left(\begin{array}{c} v_{9}^{0}\\ \omega_{9}^{0} \end{array}\right)=\left(\begin{array}{c} \sum_{i=0}^{8}\left(\left[\left(R_{i}^{0}k\right)\times \right]\left(T_{9}^{0}-T_{i}^{0}\right)\omega_{i}\right)\\ \sum_{i=0}^{8}R_{i}^{0}\omega_{i} \end{array}\right)\\ \;\;\;\;=\left(\begin{array}{c} \begin{array}{ccc} \left[\left(R_{0}^{0}k\right)\times \right]\left(T_{9}^{0}-T_{0}^{0}\right) & \cdots & \left[\left(R_{8}^{0}k\right)\times \right]\left(T_{9}^{0}-T_{8}^{0}\right) \end{array}\\ \begin{array}{ccc} R_{0}^{0}k & \cdots & R_{8}^{0}k \end{array} \end{array}\right)\left(\begin{array}{c} \omega_{0}\\ \omega_{1}\\ \vdots \\ \omega_{8} \end{array}\right)\\ \;\;\;\;=\left(\begin{array}{c} J_{v}^{9}\\ J_{\omega }^{9} \end{array}\right)\dot{q}=J\dot{q} \end{array}$$

### 2.2. Dynamics

Due to the limitation of the launching mass, the mass of the space robot will be relatively light, and the joint and its accessories will be relatively heavy, meaning the mass center of the joint and the connecting link can be placed at the joint. The Lagrange equation is used to derive the dynamic model. For a space robot, its kinetic energy is the total energy.

Kinetic energy is divided into rotational kinetic energy and translational kinetic energy. For link *i*, its kinetic energy is

(11) $$T_{i}=\frac{1}{2}m_{i}{\left(v_{i}^{1}\right)}^{T}v_{i}^{1}+\frac{1}{2}{\left(\omega_{i}^{1}\right)}^{T}I_{i}\omega_{i}^{1}$$

It can be seen from the previous kinematic analysis that

(12) $$\begin{array}{c} v_{i}^{1}=J_{v}^{i}\dot{q}\\ \omega_{i}^{1}=J_{\omega }^{i}\dot{q} \end{array}$$

which is

(13) $$\begin{array}{c} T_{i}=\frac{1}{2}m_{i}{\left(J_{v}^{i}\dot{q}\right)}^{T}J_{v}^{i}\dot{q}+\frac{1}{2}{\left(J_{\omega }^{i}\dot{q}\right)}^{T}I_{i}J_{\omega }^{i}\dot{q}\\ \;\;\;\;=\frac{1}{2}{\dot{q}}^{T}\left(m_{i}{\left(J_{v}^{i}\right)}^{T}J_{v}^{i}+{\left(J_{\omega }^{i}\right)}^{T}I_{i}J_{\omega }^{i}\right)\dot{q}\\ \;\;\;\;\;=\frac{1}{2}{\dot{q}}^{T}\left(m_{i}{\left(J_{v}^{i}\right)}^{T}J_{v}^{i}+{\left(J_{\omega }^{i}\right)}^{T}R_{i}^{1}I_{i}^{i}{\left(R_{i}^{1}\right)}^{T}J_{\omega }^{i}\right)\dot{q} \end{array}$$

where *I_ii_* is the inertia matrix of the connecting link in this system. Then, the total kinetic energy of the robot arm is

(14) $$\begin{array}{c} T=\frac{1}{2}{\dot{q}}^{T}\left(\sum m_{i}{\left(J_{v}^{i}\right)}^{T}J_{v}^{i}+{\left(J_{\omega }^{i}\right)}^{T}R_{i}^{1}I_{i}^{i}{\left(R_{i}^{1}\right)}^{T}J_{\omega }^{i}\right)\dot{q}=\frac{1}{2}{\dot{q}}^{T}M\dot{q}\\ \;\;\;=\frac{1}{2}\sum_{i=1}^{n}\sum_{j=1}^{n}m_{ij}{\dot{q}}_{i}{\dot{q}}_{j} \end{array}$$

where *M* is the generalized mass matrix of the system and a positive definite symmetric matrix; *m_ij_* is an element in *M*.

From the Lagrange equation, we can obtain

(15) $$\frac{d}{dt}\left(\frac{\partial L}{\partial {\dot{q}}_{k}}\right)-\frac{\partial L}{\partial q_{k}}=\frac{d}{dt}\left(\frac{\partial T}{\partial {\dot{q}}_{k}}\right)-\frac{\partial T}{\partial q_{k}}=Q_{k}$$

Then,

(16) $$\begin{array}{l} \frac{d}{dt}\left(\frac{\partial T}{\partial {\dot{q}}_{k}}\right)=\frac{d}{dt}\left(\sum_{j=1}^{n}m_{kj}{\dot{q}}_{j}\right)=\left(\sum_{j=1}^{n}m_{kj}\frac{d{\dot{q}}_{j}}{dt}\right)+\left(\sum_{j=1}^{n}\frac{dm_{kj}}{dt}{\dot{q}}_{j}\right)\\ =\sum_{j=1}^{n}m_{kj}{\ddot{q}}_{j}+\sum_{i=1}^{n}\sum_{j=1}^{n}\frac{\partial m_{kj}}{\partial q_{i}}{\dot{q}}_{i}{\dot{q}}_{j}\\ \frac{\partial T}{\partial q_{k}}=\frac{1}{2}\frac{\sum_{i=1}^{n}\sum_{j=1}^{n}m_{ij}{\dot{q}}_{i}{\dot{q}}_{j}}{\partial q_{k}}=\frac{1}{2}\sum_{i=1}^{n}\sum_{j=1}^{n}\frac{\partial m_{ij}}{\partial q_{k}}{\dot{q}}_{i}{\dot{q}}_{j} \end{array}$$

Thus,

(17) $$\sum_{j=1}^{n}m_{kj}{\ddot{q}}_{j}+\frac{1}{2}\sum_{i=1}^{n}\sum_{j=1}^{n}\left(\frac{\partial m_{kj}}{\partial q_{i}}+\frac{\partial m_{ki}}{\partial q_{j}}-\frac{\partial m_{ij}}{\partial q_{k}}\right){\dot{q}}_{i}{\dot{q}}_{j}=Q_{k}$$

Therefore, the dynamic equation of the robot arm is

(18) $$\left(\begin{array}{cccc} m_{k1} & m_{k2} & \cdots & m_{kn} \end{array}\right)\left(\begin{array}{c} {\ddot{q}}_{1}\\ \vdots \\ {\ddot{q}}_{n} \end{array}\right)+\frac{1}{2}\left(\begin{array}{cccc} c_{k1} & c_{k2} & \cdots & c_{kn} \end{array}\right)\left(\begin{array}{c} {\dot{q}}_{1}\\ \vdots \\ {\dot{q}}_{n} \end{array}\right)=Q_{k}$$

where

(19) $$c_{kj}=\sum_{i=1}^{n}\left(\frac{\partial m_{kj}}{\partial q_{i}}+\frac{\partial m_{ki}}{\partial q_{j}}-\frac{\partial m_{ij}}{\partial q_{k}}\right){\dot{q}}_{i}$$

## 3. Space Detumbling Robot Arm Path Planning Based on Bi-FMT* Algorithm

### 3.1. Bi-FMT* Algorithm

The input of the FMT* algorithm is the set *S* of the initial state *X*_initial_, the target state *X*_goal_ and all sampling points *X*_S_ in the free state space *X*_free_. Assume that when the Euclidean distance of two sampling points satisfies Equation (20), we state that these two sampling points are adjacent.

(20) $$\Delta x<r_{n}=\left(2+\eta \right){\left(\frac{1}{d}\right)}^{\frac{1}{d}}{\left(\frac{\mu \left(X_{free}\right)}{V_{d}}\right)}^{\frac{1}{d}}{\left(\frac{\log\left(n\right)}{n}\right)}^{\frac{1}{d}}$$

**Figure 3:**
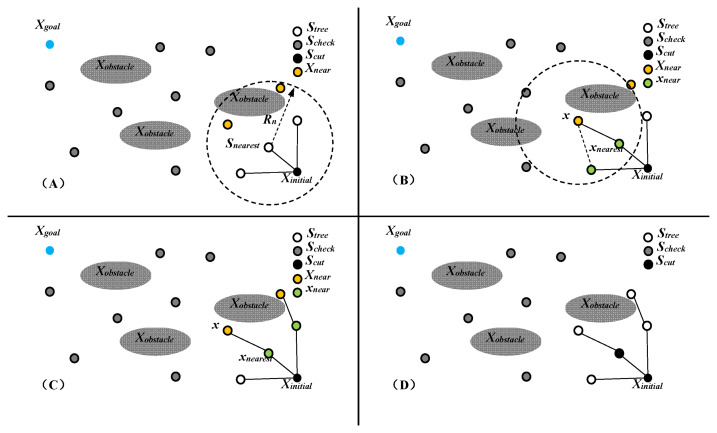
Schematic diagram of the iterative process of the FMT* algorithm. Sub-figure (**A**) shows the neighborhood point *X_near_*. Sub-figure (**B**) shows the connection of a new branch. Sub-figure (**C**) shows the all branches added based on *X_near_*. Sub-figure (**D**) shows the one step result of FMT*.

Among them, *n* is the number of sampling points, *d* is the dimension of the state space, *η* is the neighborhood radius coefficient, which is generally greater than 0, *μ*(⋅) is the Lebesgue measure and *V_d_* represents the unit sphere volume in a *d*-dimensional space. The basic description of the FMT* algorithm is shown in Figure 3. 

In the FMT* algorithm, the set *S* is divided into three subsets: the node set *S_tree_* of the tree, the candidate point set *S_check_* and the pruning set *S_cut_*. *S_tree_* includes sampling points that have been added to the path tree and continue to participate in the next step of the path tree growth; *S_check_* includes all sampling points that have not been tested; *S_cut_* includes sampling points that have been added to the path tree but are pruned during the growth of the tree The next sampling point no longer participates in the further growth of the path tree. At the beginning, the FMT* algorithm puts *X_initial_* in *S_tree_* and all other sampling points in *S_check_*, while *S_cut_* is an empty set; then, the algorithm finds the shortest point in *S_tree_* from the *X_initial_* path *S_nearest_* and finds *S_nearest_* in *S_check_*. The neighborhood point *X_near_* is shown in Figure 3A; for each sampling point x in *X_near_*, in turn, find its neighborhood point x_near_ in *S_tree_*, evaluate the path cost of each connection and find the node with the lowest path cost *x_nearest_*; if this connection path does not conflict with the obstacle area, it is added as a branch of the tree, as shown in Figure 3B; when each x in *X_near_* has completed the above operation, as shown in Figure 3C, move the sampling points successfully connected to the tree from *S_check_* to *S_tree_*, and at the same time, move *S_nearest_* from *S_tree_* to *S_cut_*, and no longer participate in the growth of the tree, as shown in Figure 3D; keep repeating the above steps to let the tree grow until *S_tree_* includes *X_goal_* or *S_tree_* is an empty set, and the algorithm ends.

**Figure 4:**
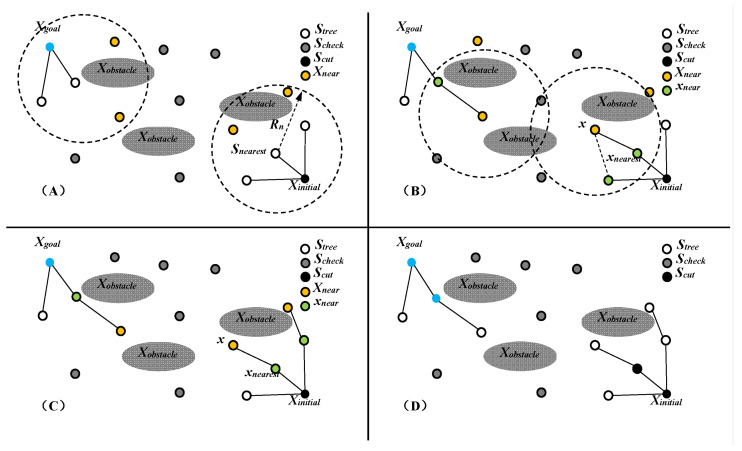
Schematic diagram of the iterative process of the Bi-FMT* algorithm. Sub-figure (**A**) shows the neighborhood point *X_near_*. Sub-figure (**B**) shows the connection of a new branch. Sub-figure (**C**) shows the all branches added based on *X_near_*. Sub-figure (**D**) shows the one step result of Bi-FMT*.

It can be seen that the FMT* algorithm synchronizes the construction and search of the path graph. By transforming the collision detection into a local optimal connection problem, a large number of collision detections are avoided. While reducing the computational complexity, it can also ensure the result of path planning. In order to improve the convergence speed of the algorithm, some scholars [60] applied the bidirectional search idea to path planning, trying to search from the initial state to the target state and from the target state back to the initial state. Studies have found that the convergence speed can be greatly improved through bidirectional planning, and this idea can basically be applied to any path planning problem [61,62]. The basic description of the Bi-FMT* algorithm is shown in Figure 4. 

The core of the Bi-FMT* algorithm is bidirectional. Except for two growing trees, the Bi-FMT* algorithm is basically the same as the FMT* algorithm. Although the basic structure of the Bi-FMT* algorithm is the same as that of the FMT* algorithm, its calculation efficiency on a given sample is much higher than that of the FMT* algorithm. When the dimension of the state space is d, the speed of the Bi-FMT* algorithm can be increased by 2^d−1^ times compared with the FMT* algorithm.

### 3.2. Problem Definition

The state space *Θ* is defined as the rotation angles {*θ*_1_, *θ*_2_, *θ*_3_, *θ*_4_, *θ*_5_, *θ*_6_} of the manipulator joints. *Θ_initial_* denotes the initial configuration of the robot arm, and *Θ_goal_* denotes the goal configuration. The manipulator path planning is expressed as follows.

Given:*Θ*_*initial*_, *t*_0_, *Θ*_*goal*_, Θ_*free*_Cost function:

$$J(\Theta (t))=tr(JJ^{T})$$

Constraints:

$$\begin{array}{c} \Theta (t_{0})=\Theta_{\mathrm{initial}}\\ \Theta (t_{f})=\Theta_{\mathrm{goal}}\\ t_{0}\;<\;t_{f}\\ g(\Theta (t),\;\tau (t),t)\;\leq \;0\\ h(\Theta (t),\;\tau (t),t)\;=\;0 \end{array}$$



### 3.3. Constraint Analysis

#### 3.3.1. Time Constraints

This paper assumes that the running time between adjacent path points is equal, that is,

(21) $$t_{f}-t_{N}=t_{N}-t_{N-1}=\cdots t_{2}-t_{1}=t_{1}-t_{0}=\Delta t$$

In addition, the total time the robot moves from the initial state to the target state should be within the time required by the task, i.e.,

(22) $$t_{f}-t_{0}\leq T_{plan}$$

The longer the response time, the smaller the required angular acceleration, the more stable the movement of the robot arm and the smoother the trajectory, the longer the travel time. DELTA.t between adjacent path points can be lengthened as much as possible during planning. From Formulas (21) and (22), the maximum value of Δ*t* is found:

(23) $$\Delta t_{\max}=\frac{T_{plan}}{N+1}$$

In this paper, Δ*t* is taken as the maximum value Δ*t*_max_, which is involved in subsequent path planning.

#### 3.3.2. Stationary Constraints

The motion of the manipulator should be smooth, and unsteady motion will intensify the relative motion between the components and cause system vibration and impact. Therefore, in addition to describing the continuity of the function of the motion trajectory of the manipulator, its velocity and acceleration should be continuous.

At this time, the path sampling point *Θ*(*t_i_*) satisfies

(24) $$\begin{array}{l} \Theta \left(t_{i}-\right)=\Theta \left(t_{i}\right)=\Theta \left(t_{i}+\right)\\ \dot{\Theta }\left(t_{i}-\right)=\dot{\Theta }\left(t_{i}+\right)\\ \ddot{\Theta }\left(t_{i}-\right)=\ddot{\Theta }\left(t_{i}+\right) \end{array}$$

where *Θ*(*t_i_*−) is a local planning trajectory before time *t_i_*, and *Θ*(*t_i_*+) is the local planning trajectory after time *t_i_*.

#### 3.3.3. Dynamic Characteristic Constraints

The dynamic characteristic constraint mainly means that the angular velocity and angular acceleration of the joint satisfy the bounded condition

(25) $$\begin{array}{l} \left|\dot{\Theta }\left(t\right)\right|\leq \omega_{\max}\\ \left|\ddot{\Theta }\left(t\right)\right|\leq {\dot{\omega }}_{\max} \end{array}$$

### 3.4. Path Planning Algorithm Description

The robot path planning algorithm is described in Table 3.

## 4. Simulation

### 4.1. Robot Model

ADAMS and MATLAB were used to simulate the model, and the MATLAB results were compared with the ADAMS results to verify the correctness of the model. Combining common components and their characteristics, the simulation parameter settings of the space detumbling robot are shown in Table 4.

DH parameters are shown in Table 5.

**Figure 5:**
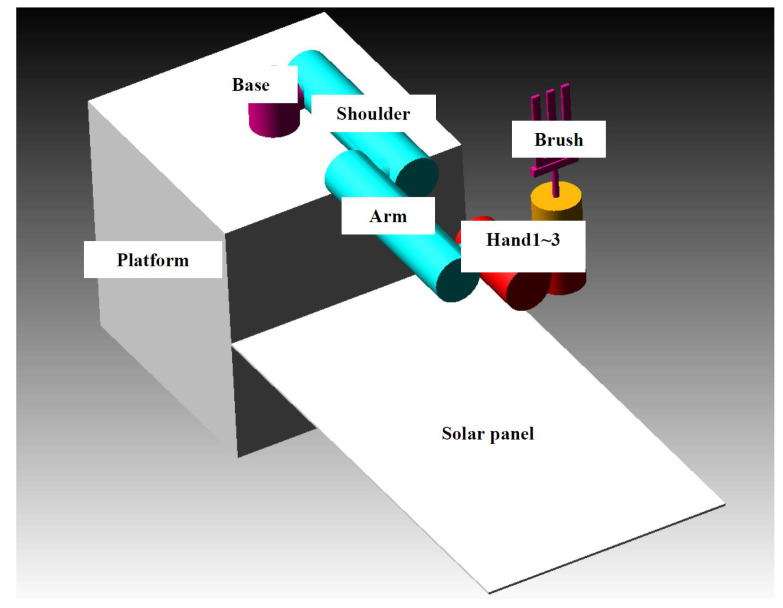
Configuration of the space detumbling robot.

**Figure 6:**
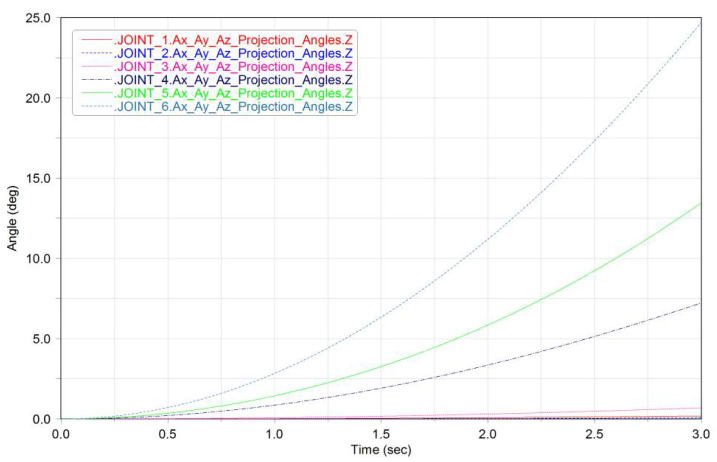
Diagram of the rotation angle of each joint over time (ADAMS).

**Figure 7:**
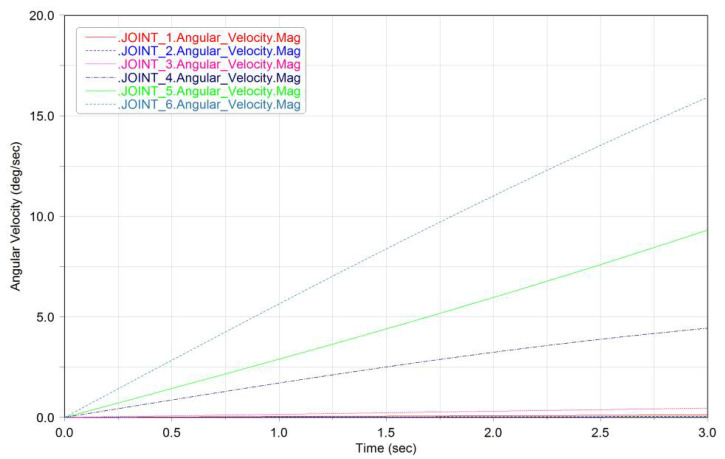
The angular velocity of each joint over time (ADAMS).

We constructed the space detumbling robot model in ADAMS, as shown in Figure 5. Under the torque applied by the joints, the robot simulation results are shown in Figure 6 and Figure 7.

**Figure 8:**
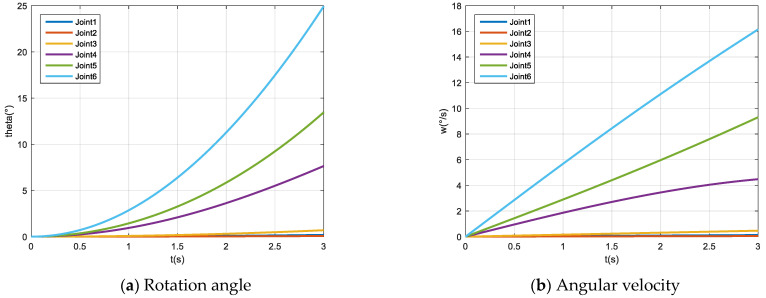
MATLAB simulation results.

According to the simulation parameters, the kinematics and dynamics equations of the robot are specifically expanded (see Appendix B of [63] for details). We used MATLAB to build the model, import the simulation parameters and obtain the simulation results, as shown in Figure 8.

**Figure 9:**
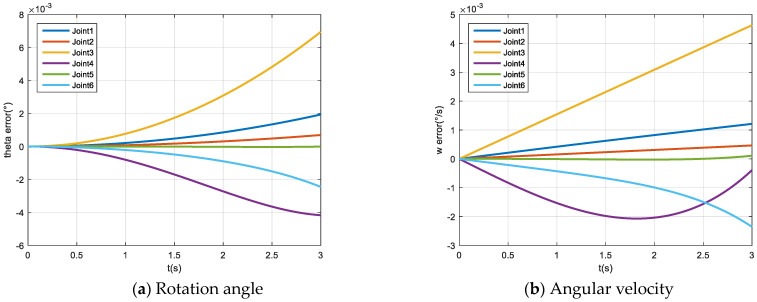
Deviation vs. time graph.

We compared the ADAMS calculation results with the MATLAB mathematical model simulation results, as shown in Figure 9. It can be seen from the figure that the simulation results’ deviation is small, which verifies the correctness of the robot model.

### 4.2. Path Planning

**Figure 10:**
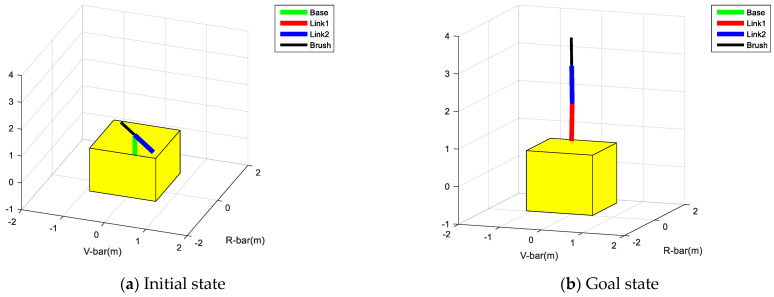
Schematic diagrams of initial and goal states.

The initial and goal states of the robot are shown in Figure 10.

$$\begin{array}{l} \Theta_{initial}=\left(\begin{array}{cccccc} \frac{\pi }{4} & 0 & \pi & 0 & \frac{\pi }{2} & 0 \end{array}\right)\\ \Theta_{goal}=\left(\begin{array}{cccccc} 0 & \frac{\pi }{2} & 0 & 0 & \frac{\pi }{2} & 0 \end{array}\right) \end{array}$$

It should be noted that the arm wrist joint can be locked, and thus the sampling state space can be expressed as

(26) $$\Theta =\left(\begin{array}{cccccc} \theta_{1} & \theta_{2} & \theta_{3} & 0 & \frac{\pi }{2} & 0 \end{array}\right)$$

On the other hand, under this simplified condition, the non-collision condition can be expressed as

(27) $$\begin{array}{l} 0\leq \theta_{2}\leq \pi \\ -2\theta_{2}\leq \theta_{3}\leq \pi \end{array}$$

Considering that the flexible brush has a length, the state of the manipulator must also be satisfied:

(28) $$0\leq \theta_{2}+\theta_{3}\leq \pi$$

In summary, the sampling state space is

(29) $$\Theta =\left\{\begin{array}{cc} \left(\begin{array}{cccccc} \theta_{1} & \theta_{2} & \theta_{3} & 0 & \frac{\pi }{2} & 0 \end{array}\right) & |\begin{array}{l} 0\leq \theta_{2}\leq \pi \\ 0\leq \theta_{2}+\theta_{3}\leq \pi \end{array} \end{array}\right\}$$

while

$$\begin{array}{l} T_{plan}=600\;s\\ N=1000\\ \omega_{\max}=0.5{}^{{}^{\circ}}/s\\ {\dot{\omega }}_{\max}=5{}^{{}^{\circ}}/s^{2} \end{array}$$

The data structure defining the sampling points in the robot path planning is as follows:

(30) $$\Theta_{i}=\left\{\begin{array}{ccccccc} i & ¦\begin{array}{cccccc} \theta_{1} & \theta_{2} & \theta_{3} & \theta_{4} & \theta_{5} & \theta_{6} \end{array} & ¦iftoinitial & ¦iftogoal & ¦j & ¦J_{ij} & ¦\begin{array}{cc} J_{initial} & J_{goal} \end{array} \end{array}\right\}$$

where *i* is the sample point number; *iftoinitial* and *iftogoal* are identifiers for determining that the node is connected to the initial state or the goal state; *j* denotes the node from the node to the initial connection point.

**Figure 11:**
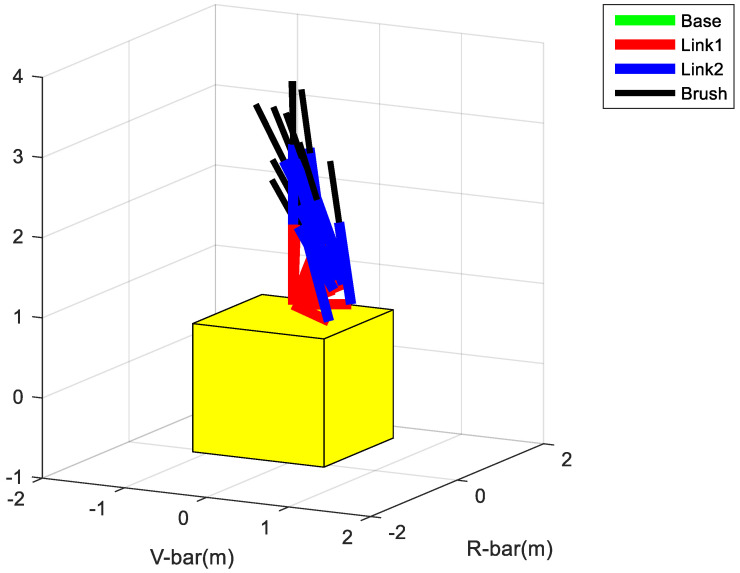
Path planning results.

**Figure 12:**
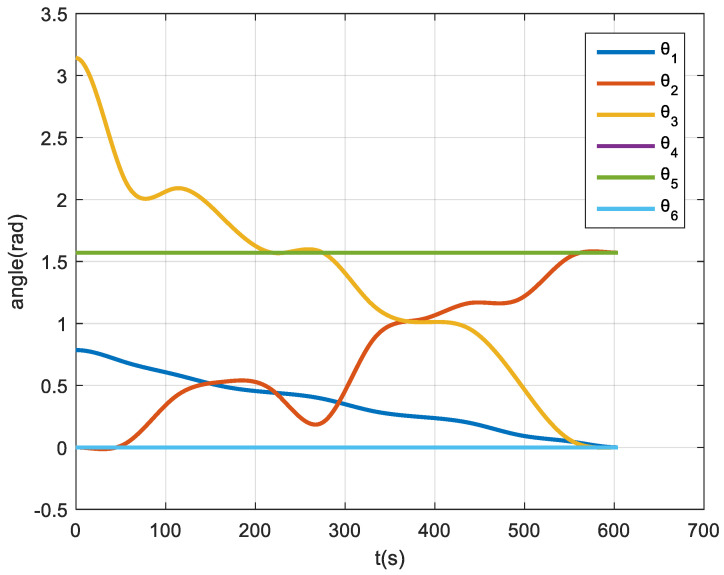
Manipulator trajectory planning results.

The hardware conditions are Intel(R)Core(TM)i7-4720HQ CPU@2.60GHz, 8.00GB RAM. The simulation platform adopts MATLAB2015b. The simulation results are shown in Figure 11. Under the hardware conditions presented in this paper, the path planning time is 0.567 s when the sampling number is 1000. It can be seen from the figure that the robot arm can reach the goal configuration, which shows the validity of the robot arm path planning algorithm. Considering that the increase in sampling points makes the solution matrix very large, this paper adopted a two-step strategy: first, according to the full sampling space to carry out the path planning, we generated the sequence of path sampling points; then, we selected a small sample to generate the trajectory in the obtained sequence. Taking Figure 11 as an example, the resulting trajectory equation is shown in Figure 12.

## 5. Conclusions and Future Work

With the development of space exploration technology and space commercial activities, the number of spacecrafts in space is sharply increasing, and space resources and the environment are facing enormous challenges. On-orbit service (OOS, which consists of on-orbit refueling, on-orbit repairing, on-orbit upgrading and space debris removal) is an effective means to achieve successful space exploration missions and keep the space environment safe. Whether on-orbit assembly or space debris removal, the proximity to non-cooperative targets is important. However, these non-cooperative targets usually have complex attitude movements, which greatly affect the proximity operation process. In order to avoid damage to service satellites and targets during operation and improve safety and reliability, it is necessary to study how to eliminate or reduce the rotation of targets. A series of technical verification tests have been conducted by the space powers, proposing numerous detumbling methods, including friction, static, net, auxiliary device and electromagnetic. Considering technical maturity and energy consumption, among these methods, frictional detumbling is the most feasible. This paper focused on a space detumbling robot and studied the related technologies including space detumbling robot dynamics and robot arm path planning. A certain space detumbling robot with a ‘platform + manipulator + end effector’ configuration was proposed. By considering the end effector as a translational joint, the kinematic and dynamic model of the space detumbling robot was presented. Then, ADAMS and MATLAB were used to simulate and verify the model. After that, the robot arm deployment problem was analyzed in detail, and path planning based on the Bi-FMT* algorithm was also proposed and verified by simulation. 

Space detumbling is a multi-disciplinary complex system engineering problem involving basic disciplines such as mathematics, physics and materials and combining technical disciplines such as control, computer and simulation. In contrast, the research work conducted in this article is only a small part of the solution, there is still a big gap to fill before practical engineering applications and theoretical research needs to be improved. On the basis of this article, future work directions include the following:

(1) The platform, manipulator and target are all regarded as rigid bodies; in practice, both the manipulator and solar panels have a certain degree of flexibility, and modeling under the condition of multiple flexible bodies is an important research direction.

(2) Semi-physical design and simulation verification of detumbling platforms and mechanisms.

## Figures and Tables

**Table 1:** Summary of the detumbling methods in recent years.

Category	Schematic Diagram	Brief Description
Injection [4,5,6,7,8]	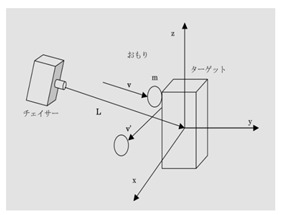	The service satellite injects substances such as a gas, ion beam or laser into the target, changing the quality characteristics of the target, including mass and inertia. Thus, it is known from the angular momentum conservation law that the target will be detumbling. On the other hand, the injection could hinder the movement of the target, thereby achieving the purpose of eliminating the target rotation. Using this method, it is necessary to carry an additional end effector and substances for the purpose of detumbling, except for gas injection which can be injected through its own engine but needs more fuel.
Auxiliary Device [9,10,11]	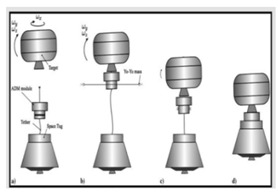	Attaching an auxiliary device to the target through the service satellite and using the auxiliary device to eliminate the target rotation. The service satellite can avoid contact with the target, and the detumbling mode can flexibly adopt various means according to the target situation. However, similar to the above, the detumbling mode needs to be an additional device that is dedicated to the service satellite and has certain maneuverability and controllability which increases the system complexity.
Electrostatic [12,13,14,15]	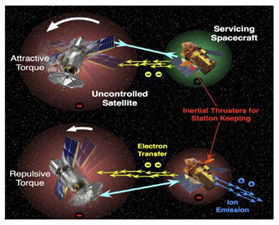	Electrons are continuously emitted to the target through an electron-emitting device carried on the service satellite, charging the target. By doing this, the target rotation is detumbled by Coulomb electrostatic force generated by the voltage difference between the service satellite and the target. This also avoids contact between the service satellite and target. However, in this method, it is necessary to continuously charge and discharge to change the potential of the service satellite and target. In addition, this method needs to be further studied in the identification of target charge and discharge characteristics, formation maintenance and charge and discharge control algorithms.
Electromagnetic [16,17,18,19,20]	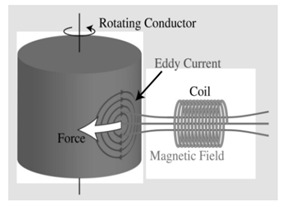	Space debris mostly contains conductive materials such as aluminum alloys and titanium alloys. Therefore, when the target is in an external magnetic field, eddy currents are internally induced to hinder the relative motion. By using a superconducting coil to construct an external magnetic field, the target can be detumbled. The electromagnetic damping effect passively eliminates the component angular rate perpendicular to the component of the external magnetic field but does not affect the angular rate component parallel to the magnetic field direction. Thus, the relative position between the magnetic field source and the target is to be changed. In addition, the use of superconducting coils to construct a wide range of electromagnetic fields requires a corresponding power supply and cooling system. How to superimpose the superconducting magnetic field source system with service satellites requires further study.
Robotic Contact [21,22,23]	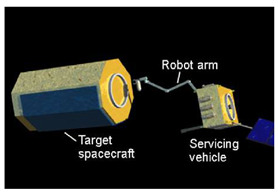	In this method, the service satellite touches the target intermittently by using the elastic deceleration device attached to the end of the arm. The target rotation is detumbled by the friction. Robotic contact detumbling can actively adjust the direction, size and time of the control force and provide a higher braking efficiency with an accurate torque control model. However, this type of detumbling mode needs a service satellite to perform a complex orbit maneuver before implementation, located at a position very close to the target, and the collision risk is also increased. In addition, it is suitable for a target with a lower speed considering the on-orbit identification efficiency and the manipulator control precision.
Net or Tether [24,25,26,27,28]	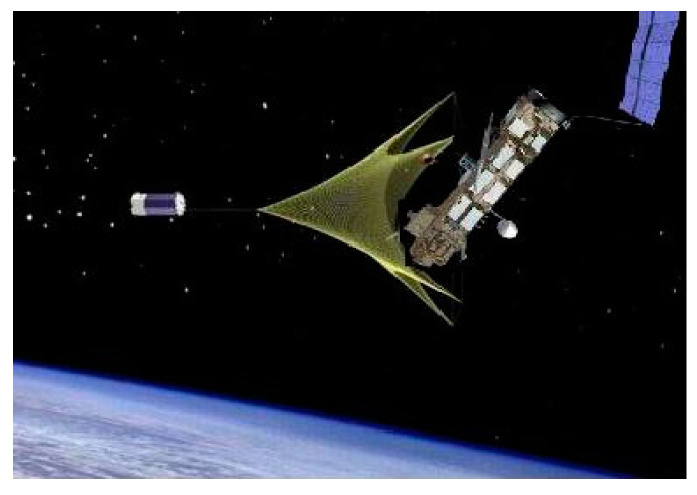	When the net or tether catches the target, the target rotational speed is reduced by the tension and damping force of the tether. This method is only used for debris. In addition, how to avoid failure in catching and preventing the entanglement of the rope also needs further research.

**Table 2:** DH parameters of the robot.

Joint	*a_i_*	*α_i_*	*d_i_*	*θ_i_*
0	0	0	*d*_0_	0
1	0	90°	0	*θ*_1_ *
2	*L*_1_	0	0	*θ*_2_ *
3	*L*_2_	0	0	*θ*_3_ *
4	0	90°	0	*θ*_4_ *
5	0	90°	0	*θ*_5_ *
6	0	0	*d*_6_	*θ*_6_ *
7	0	0	*d*_7_ *	0
8	*a*_8_ *	0	0	0

* represents system variables.

**Table 3:** Manipulator path planning based on the Bi-FMT* algorithm.

1:	given initial state *Θ_initial_*, *Θ_goal_*, task time *T_plan_*, sampling point number *N*, maximum angular velocity *ω_max_* and maximum angular acceleration ${\dot{\omega }}_{\max}$
2:	Calculating single step response time Δ*t* by using (23)
3:	The state space is sampled by means of Halton sampling method, and the set of sampling points *Θ_S_* is obtained.
4:	The path trees {*S_tree_*, *S_check_*, *S_cut_*} and {*S’_tree_*, *S’_check_*, *S’_cut_*} which are based on *Θ_initial_* and *Θ_goal_* is generated
--	While Do
5:	Finding the intersection *S_m__eet_* of *S_tree_* and *S’_tree_*
6:	*S_meet_ is not empty*
7:	Calculate the path cost J of each point
8:	Find the sampling point *θ_meet_* with the smallest J
9:	By connecting *S_tree_* and *S’_tree_* with *θ_meet_* as the connection point, the path between *Θ_initial_* and *Θ_goal_* is obtained
10:	*S_meet_ is empty*
11:	performing FMT*algorithms on {*S_tree_*, *S_check_*, *S_cut_*} and {*S’_tree_*, *S’_check_*, *S’_cut_*}, respectively, and updating *S_tree_* and *S’_tree_*
--	While Done
12:	The local cubic polynomial interpolation is used to generate trajectory between sampling points in path

**Table 4:** List of simulation parameters of the space detumbling robot.

Type	Subtype	Value
Satellite platform	Central body	$\begin{array}{c} m=1000\;\mathrm{kg}\\ \;I=diag\left\{400,400,400\right\}\mathrm{kg}\cdot m^{2}\\ V=1.6\;m\times 1.6\;m\times 1.6\;m \end{array}$
	Solar panel	$\begin{array}{c} m=20\;\mathrm{kg}\\ \;I=diag\left\{20,1,20\right\}\mathrm{kg}\cdot m^{2}\\ V=2\;m\times 1.6\;m\times 0.015\;m \end{array}$
Robot arm	Joint1, Joint4~6	$\begin{array}{c} m=10\;\mathrm{kg}\\ \;I=diag\left\{1,1,1\right\}\mathrm{kg}\cdot m^{2}\\ V=0.3\;m\times \left(\pi \times 0.15\;m\times 0.15\;m\right) \end{array}$
	Joint2~3	$\begin{array}{c} m=10\;\mathrm{kg}\\ \;I=diag\left\{1,1,1\right\}\mathrm{kg}\cdot m^{2}\\ V=0.3\;m\times \left(\pi \times 0.15\;m\times 0.15\;m\right)\\ L=1\;m \end{array}$
Flexible brush	Flexible brush	L=0.75 m
Simulation parameters	Initial joint state	$\left[\begin{array}{cccccc} \theta_{1} & \theta_{2} & \theta_{3} & \theta_{4} & \theta_{5} & \theta_{6} \end{array}\right]=\left[\begin{array}{cccccc} 0^{\circ } & 0^{\circ } & 0^{\circ } & 90^{\circ } & 90^{\circ } & 0^{\circ } \end{array}\right]$
	Joint torque	$\left[\begin{array}{cccccc} \tau_{1} & \tau_{2} & \tau_{3} & \tau_{4} & \tau_{5} & \tau_{6} \end{array}\right]=\left[\begin{array}{cccccc} 0.1 & 0.1 & 0.1 & 0.1 & 0.1 & 0.1 \end{array}\right]N\cdot m$

DH parameters are shown in Table 5.

**Table 5:** DH parameters of the space detumbling robot.

Joint	*a_i_*	*α_i_*	*d_i_*	*θ_i_*
0	0	0	0.95	0
1	0	90°	0	90°
2	1	0	0	0
3	1	0	0	0
4	0	90°	0	90°
5	0	90°	0	90°
6	0	0	0	0
7	0	0	0.75	0
8	0	0	0	0

## Data Availability

Data is contained within the article.
